# Enhancing patient satisfaction in COVID-19 wards: a randomized controlled trial on the impact of supportive educational programs in Najaf hospitals

**DOI:** 10.3389/fpsyt.2025.1544384

**Published:** 2025-06-18

**Authors:** Dhuha Ahmed Al-Qaseer, Mohammad Namazinia, Fatemeh Hajiabadi, Seyyed Reza Mazloum, Ali A. Al-Fahham

**Affiliations:** ^1^ Department of Medical – Surgical Nursing, School of Nursing and Midwifery (MSC Student), Mashhad University of Medical Sciences, Mashhad, Iran; ^2^ Department of Nursing, School of Nursing and Midwifery, Torbat Heydariyeh University of Medical Sciences, Torbat Heydariyeh, Iran; ^3^ Department of Medical Surgical Nursing, School of Nursing and Midwifery, Mashhad University of Medical Sciences, Mashhad, Iran; ^4^ Nursing and Midwifery Care Research Center, Mashhad University of Medical Sciences, Mashhad, Iran; ^5^ Faculty of Nursing, The University of Kufa, Najaf, Iraq

**Keywords:** COVID-19, patient satisfaction, supportive educational program, randomized clinical trial, psychological support, healthcare quality

## Abstract

**Background:**

The COVID-19 pandemic has severely impacted global health, pervasively affecting the physical and mental wellbeing of individuals worldwide. As the pandemic continues, evaluating patient satisfaction within healthcare has become increasingly critical. This study examines the impact of supportive educational programs on patient satisfaction in COVID-19 wards in Najaf hospitals.

**Method:**

A randomized clinical trial involving 60 patients admitted to COVID-19 departments was conducted. The intervention group received a comprehensive supportive educational program upon hospital admission, while the control group received standard care. Participant satisfaction levels were measured using a translated and adapted version of Wolf’s Patient Satisfaction Instrument. Analysis was performed on demographic data and satisfaction scores through descriptive statistics and inferential tests using SPSS version 21.

**Results:**

The study revealed that the intervention group reported significantly higher satisfaction scores compared to the control group across all measured domains, including professional-technical care, trust, and patient education. These results suggest that supportive educational programs can significantly enhance patient satisfaction during hospitalization for COVID-19.

**Conclusion:**

Supportive educational interventions are effective in improving patient satisfaction, which is an important metric for healthcare quality. This study indicates that supplementing standard care with educational and emotional support benefits patients, pointing toward the need for integrated care approaches that address both physical and psychological needs during pandemics. Future research could focus on long-term impacts and explore the potential for virtual implementation of similar programs.

**Clinical trial registration:**

https://irct.behdasht.gov.ir/trial/58407, identifier IRCT20140625018231N1.

## Introduction

The emergence and pandemic of SARS-CoV-2 (COVID-19) in December 2019 have led to unprecedented changes in global lives and have had profound consequences for both their physical and mental health (1, 2). According to reports, as of January 16th, 2022, the disease has spread to 230 countries, with a total of 326,057,106 confirmed cases and 5,545,043 deaths; in Iraq, there have been reported 2,117,175 cases and 24,981 deaths by the mentioned date (3).

This disease is a highly contagious one that affects a vast population in a short period (4, 5). Symptoms of the virus infection include fever, chills, cough, sore throat, myalgia, nausea, vomiting, and diarrhea (6, 7). Patients with severe and critical illness require hospitalization and precise monitoring and care (8).

Besides the physical impacts, COVID-19 can have serious effects on mental health. A wide range of psychological consequences has been observed during the virus outbreak on individual, social, national, and international levels (6). On the other hand, the need for strict isolation and social distancing for COVID-19 patients, while necessary and inevitable, leads to the separation of the patient from the family and close ones who are the potential sources of psychological and social support during the illness and severe conditions, exacerbating the patient’s distress (9, 10). Ultimately, this stress, by activating the hypothalamic-pituitary-adrenal pathway and raising the blood levels of glucocorticoids, epinephrine, and norepinephrine, leads to severe anxiety, depression, and other psychological disorders, ultimately reducing patient satisfaction during the disease (11).

Patient satisfaction is introduced as an indicator of healthcare quality. This indicator reflects not only the quality of care provided but also an individual’s expectations of care (12, 13, 27, 28). Thus, today, the importance of measuring patient satisfaction as one of the most crucial and fundamental criteria for determining the quality of care services is undeniable (14). Dissatisfaction with healthcare services has undesirable consequences. People’s dissatisfaction leads to their disconnection from the health system or at least not participating in providing services (15, 29). Therefore, measuring patient satisfaction is one of the most important and challenging components of care quality assessment. Patient satisfaction is typically assessed through the patient’s recent experience at the hospital. Khatatbeh et al. (2021) reported in their study that there’s a direct correlation between the social support provided by the nurses and patient satisfaction, with increased social support leading to an increase in satisfaction (16). Likewise, Bahrami et al. (2013) reported in their study that an educational communication program emphasizing the educational needs of cancer patients undergoing surgery led to increased patient satisfaction regarding pain management (17).

Considering that, based on conducted studies, educational programs on different groups including patients’ families or the patients themselves have been carried out (18), which also improved their satisfaction. In addition, since the increase in patient satisfaction is a key indicator for health policy-makers to the extent that some hospitals use the patient satisfaction index as a quality indicator, they assess their performance and compare their level with other hospitals and national and international average indices (19).Therefore, this study aims to evaluate the impact of a supportive educational program on the satisfaction of COVID-19 patients in Najaf hospitals. Using a randomized controlled trial (RCT) design, we seek to provide evidence-based strategies for improving patient-centered care during pandemics.

## Method

### Study design

**Figure 1:**
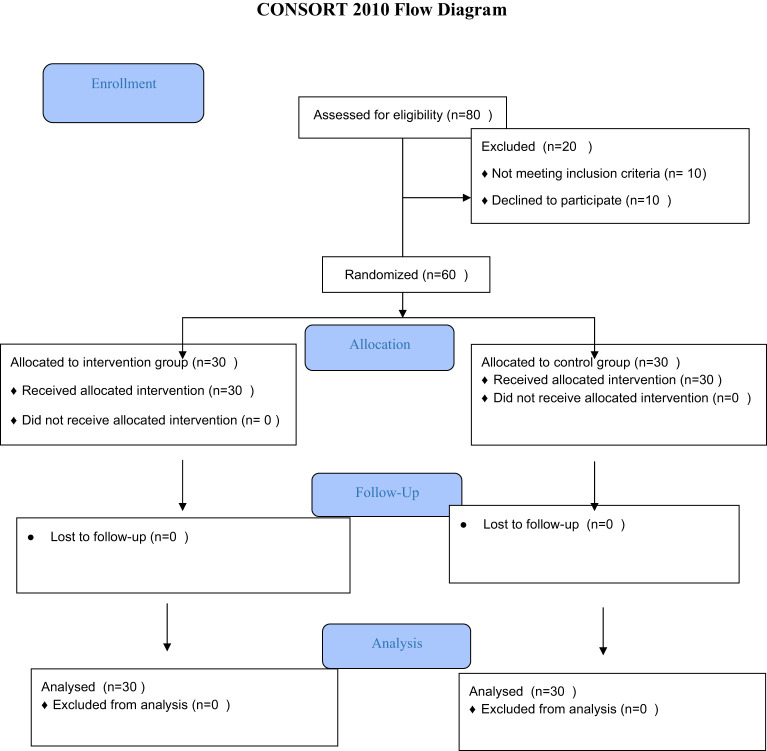
The CONSORT diagram of study.

The present study was a randomized clinical trial with a witness, with the trial code IR.MUMS.NURSE.REC.1400.028. It was designed and implemented on 60 patients attending the COVID departments of the COVID hospitals in the city of Najaf in the year 2021. The hospitals of Al-Amal, Al-Hakeem, and Al-Sadr in Najaf, which are part of educational, therapeutic, and research hospitals containing COVID sections, constituted the environment of this research (**Figure 1**).

### Participants

Inclusion criteria for the study included: age between 18 and 65 years, positive COVID PCR test, confirmed diagnosis of COVID-19, need for hospitalization, no previous infection with COVID-19, not working as a medical staff member, no auditory or visual problems, and having minimum literacy skills to read and write. Exclusion criteria included: unwillingness to continue cooperation at any stage of the research, need for intubation or tracheostomy for the patient, and any conditions leading to non-cooperation of the patient throughout the study, such as a decrease in GCS and severe respiratory or hemodynamic disorders.

### Outcomes

The instruments used in the present study included a demographic information form and the Patient Satisfaction Instrument (PSI), also known as Wolf’s Patient Satisfaction Instrument. This tool was selected for its validated reliability and relevance in assessing patient satisfaction, particularly in high-stress healthcare settings such as COVID-19 wards. Its specific focus on dimensions of patient experience aligns closely with the objectives of this study. The demographic information form, comprising several questions about gender, marital status, income, and place of residence, was designed based on previous studies and consultations with expert professors and advisors, and was considered valid due to the repetitive nature of the questions. This questionnaire was completed through an interview.

The PSI or Wolf’s Patient Satisfaction Instrument was first translated and adapted into Persian by Hajinejad in Iran (19) and then by July and colleagues minor changes were made to this questionnaire (20). The final questionnaire contains 7 items related to the sub-scale of professional-technical care, 13 items related to the trust sub-scale, and 6 items related to the educational sub-scale to patients. Each item is rated on a Likert scale, ranging from completely agree (score 5) to completely disagree (score 1), with 14 positive items and 12 negative items being reverse scored. A score of less than 78 is assessed as dissatisfied, 78 to 104 as moderate satisfaction, and over 104 as complete satisfaction. Accordingly, for the sub-scales of professional-technical care, scores of less than 21 are unhappy, between 21 to 28 moderate satisfaction, and over 28 complete satisfaction; for the trust scale, less than 39 is unhappy, 39 to 52 moderate satisfaction and more than 52 complete satisfaction; and for the educational sub-scale to patients, scores of less than 18 are unhappy, 18–26 moderate satisfaction, and over 26 complete satisfaction (19).

To determine the validity of the instruments, content validity was assessed by having the instruments reviewed by ten members of the scientific board of the Nursing and Midwifery Faculty in Mashhad and four faculty members from the Nursing School of Kufa in Najaf. After incorporating their feedback and final revisions, the instruments were utilized. The reliability of the instruments was established using Cronbach’s alpha, which was calculated by administering the questionnaire to 20 participants who met the study’s inclusion criteria. The Cronbach’s alpha coefficient for the overall scale was 0.90, with subscale coefficients of 0.91 for depression, 0.83 for anxiety, and 0.85 for stress, confirming the instrument’s high reliability.

The tool was completed 5 days after hospital admission for both the control and intervention groups.

This instrument was chosen because it offers a comprehensive evaluation of patient satisfaction by assessing three critical dimensions—professional-technical care, trust, and patient education—that are particularly relevant in the context of COVID-19 care. Its previous adaptation and validation for the Persian-speaking population, with demonstrated strong psychometric properties (Cronbach’s alpha = 0.90), further supports its suitability for our study. This instrument enables us to capture the nuanced aspects of patient satisfaction that are essential for assessing the impact of supportive educational interventions in a pandemic setting.

### Sample size and randomization

To determine the sample size, due to the lack of similar study results available, a pilot study was conducted on 10 people in each group using a mean comparison formula with a 95% confidence coefficient and an 80% test power for all study outcomes. The sample size was estimated at 25 participants per group. However, to enhance the statistical power for detecting differences in patient satisfaction—the primary outcome of this study—and to allow for subgroup comparisons, we increased the sample size by accounting for a 20% dropout rate. Thus, 30 participants were included in each group, resulting in a total sample size of 60.

The randomization of study units into intervention and control groups was achieved through a random sequence generated by the Random.org website. A concealed allocation approach was utilized using sealed envelopes; random sequences were written as codes A and B on small cards and placed inside the envelope. When a patient meeting the research unit criteria was identified, the envelope was opened, and the code inside determined the group assignment.

Regarding blinding, due to the nature of the intervention, blinding of participants and healthcare providers was not feasible. However, to minimize bias, the data collectors and statistical analysts were blinded to the group assignments. Additionally, since patients were isolated due to COVID-19 restrictions, there was no possibility of information dissemination between the two groups.

### Data collection

Data collection was conducted using standardized tools and procedures to ensure the accuracy and reliability of the gathered information. Patients’ satisfaction levels were assessed using the validated Patient Satisfaction Instrument (PSI). Data were collected at baseline (upon hospital admission) and after the intervention period through structured interviews or self-administered questionnaires, depending on patient preference and condition.

### Intervention

The supportive educational program began upon hospital admission and the confirmation of COVID-19 infection.

#### Supportive component

- Upon admission, the researcher introduced themselves to the patient, who had just been informed of their COVID-19 diagnosis and the need for hospitalization and isolation.
- The researcher provided emotional support by staying by the patient’s side, demonstrating empathy, addressing concerns, and building trust.
- A key supportive strategy involved identifying a trusted companion among the patient’s relatives and providing the patient with a contact number for daily phone and video calls during hospitalization.
- The researcher remained available during specified hours in the morning and evening for consultation and emotional support, responding to patient concerns directly or by consulting the medical team as needed.
- Daily bedside visits were conducted to provide continued support, answer emerging questions, and assess patient needs.

#### Educational component

- The researcher provided accurate information regarding the disease process, treatment, the necessity and benefits of hospitalization, and potential risks of avoiding hospitalization.
- Patients were reassured that hospitalization did not necessarily indicate a severe condition and that many hospitalized patients successfully recover and are discharged.
- A comprehensive pamphlet, developed based on the educational needs of hospitalized COVID-19 patients (as identified by the research team and literature review), was given to patients. The pamphlet covered key topics, including:
  - COVID-19 definitions and severity levels
  - Reasons for hospitalization and required treatments
  - Importance and benefits of isolation
  - Nutritional recommendations and beneficial activities during hospitalization
  - Methods for maintaining communication with family during isolation
  - A designated contact number for further consultation during hospitalization
- Education was reinforced through daily interactions, ensuring that patient concerns were addressed and additional guidance was provided as needed.

This program was designed based on an extensive literature review of the educational and supportive needs of hospitalized COVID-19 patients, as well as expert input from the research team (6, 19, 20). The control group only received usual care in the department.

#### Intervention fidelity

To ensure the fidelity of the supportive educational program, several measures were implemented:

- Prior to the start of the study, all research staff involved in delivering the intervention underwent standardized training on the intervention protocol.
- A detailed checklist was developed to outline all key components of the intervention, which was used during each session to verify that every aspect was consistently addressed.
- Regular supervisory meetings were conducted to review adherence to the protocol, and independent observers performed random assessments of selected intervention sessions.
- Any deviations identified were immediately addressed through additional training or corrective measures.

### Statistical analysis

After coding and entering the data into SPSS version 21, descriptive statistics including frequency distribution tables, means, and standard deviations were used to describe the sample characteristics. The normality of quantitative variables was assessed using the Kolmogorov-Smirnov and Shapiro-Wilk tests. Based on the results, appropriate inferential statistical tests were selected: the Chi-square test was applied to examine associations between categorical variables across groups, as it is suitable for analyzing relationships between independent qualitative variables, while the independent T-test was used to compare continuous variables between groups, given that the data met the assumption of normal distribution. All statistical analyses were performed at a 95% confidence level with a significance threshold of 0.05.

## Results

**Table 1** presents the demographic characteristics of the participants. The mean age of the intervention group was 49.1 ± 13.2 years, while that of the control group was 41.6 ± 12.8 years, showing a significant difference between the two groups (**P=0.030). In terms of gender, 53.3% of the intervention group were female and 46.7% were male, whereas in the control group, 70.0% were female and 30.0% were male (P=0.184). Regarding marital status, the majority of participants in both groups were married (62.1% in the intervention group and 60.0% in the control group), with no significant difference between the two groups (P=0.714). Additionally, most participants were urban residents (83.3% in the intervention group and 76.7% in the control group), and there was no significant difference in place of residence between the two groups (P=0.519). As for family income, the majority of participants in both groups had weak or average income (76.7% in the intervention group and 70.0% in the control group), with no significant difference observed between the two groups (P=0.559) (**Table 1**).

**Table 1:** Demographic characteristics of the participants.

Variable	Intervention N (%)	Control N (%)	P
Age (Mean ± SD)	49.1 ± 13.2	41.6 ± 12.8	**P= 0.030
Sex			
Female	16 (53.3)	21 (70.0)	*P=0.184
Male	14 (46.7)	9 (30.0)	
Marital status			
Single	6 (20.7)	8 (26.7)	*P=0.714
Married	18 (62.1)	18 (60.0)	
deceased wife	5 (17.2)	3 (10.0)	
Divorced	0 (0.0)	1 (3.3)	
Place of home			
city	25 (83.3)	23 (76.7)	*P=0.519
village	5 (16.7)	7 (23.3)	
Family income			
Weak or average	23 (76.7)	21 (70.0)	*P=0.559
good or great	7 (23.3)	9 (30.0)	

*Chi-square.

**independent t.

The results detailed in **Table 2** highlight that the average total satisfaction score was significantly higher in the intervention group (94.8 ± 9.6) compared to the control group (87.6 ± 10.9), with a P-value of 0.010. Further, the professional technical care received an average score of 47.9 ± 4.7 in the intervention group, markedly greater than the control group’s 41.9 ± 6.1, with P<0.001 denoting high statistical significance. The trust dimension too reflected a similar trend, scoring an average of 88.9 ± 8.6 in the intervention group against 82.3 ± 7.9 in the control, alongside a strongly significant P-value of <0.001. Lastly, the educational dimension to the patient showcased an average score of 41.1 ± 3.9 for the intervention group, which was substantially higher than the control group’s 33.4 ± 3.5, with a P-value of <0.001 (**Table 2**).

**Table 2:** The average and standard deviation of the total satisfaction score of the studied patients during the stages by group.

Variable	Group		P
	Intervention	Control	
	(Mean ± SD)	(Mean ± SD)	
Total satisfaction score	94.8± 9.6	87.6± 10.9	*P=0.010
Professional technical care	47.9± 4.7	41.9± 6.1	*P<0.001
Trust dimension	88.9± 8.6	82.3± 7.9	*P<0.001
Educational dimension to the patient	41.1± 3.9	33.4± 3.5	*P<0.001

*independent t.

## Discussion

Discussion The present research was a two-group randomized clinical trial aimed at determining the effect of a supportive educational program on the satisfaction of COVID-19 patients admitted to COVID wards. The overall results of the study indicated the positive impact of the supportive educational program on the satisfaction of these patients.

Compared to previous studies, our intervention was uniquely designed to address the psychological, informational, and emotional needs of COVID-19 patients through a structured and comprehensive approach. While previous research has demonstrated the effectiveness of educational and supportive interventions in various patient populations, our study contributes additional value by tailoring these strategies specifically to COVID-19 patients, who faced unprecedented psychological distress and uncertainty during hospitalization.

The enhancement of patient satisfaction in our study can be attributed to several factors. First, the structured nature of the program ensured that patients received clear and relevant information, reducing uncertainty and anxiety associated with COVID-19 hospitalization. Second, the psychological support incorporated into the intervention helped patients manage stress and cope with their illness more effectively. Lastly, the interactive engagement between healthcare providers and patients fostered a sense of trust and reassurance, which has been shown to positively influence patient satisfaction.

Bahrami and associates (2013) reported in their study that an educational communication program focused on the educational needs of cancer patients undergoing surgery increased patient satisfaction with pain management (17). Similarly, Khatatbeh et al. (2021) reported that there was a direct correlation between the social support provided by nurses and parental satisfaction with the care of premature infants, indicating that increased social support led to increased parental satisfaction (16). The support provided by the nurses, by covering the scope of individuals’ needs, could lead to increased satisfaction. Therefore, the findings of this study are consistent with those of our current research.

Vatan Doust and colleagues (2015) found in their study on tracheostomy patients that the use of an instructional video, along with usual teachings, could improve the quality of life and increase patient satisfaction (21). The results of the study by Shoushi et al. (2018) also showed that implementing an educational program for family caregivers increased their satisfaction with nursing care, which aligns with the findings of the present study (22).

The study conducted by Kang and colleagues suggested that a psychological support intervention that included encouraging patients to express their feelings, demonstrating understanding and reassurance, providing knowledge and information about COVID-19, offering a few simple relaxation techniques, promoting self-management skills (such as listening to music as a distraction in a bad mood), and ultimately helping to relieve psychological tension and build confidence to overcome illness, as well as convincing them to cooperate with the medical staff and maintain an optimistic outlook, proved beneficial. These interventions were given by two physicians and lasted for 15 minutes (23). Thus, the results are in line with those of our current study.

Unlike these previous studies, our supportive educational program integrated multiple components, including structured information delivery, psychological support, and interactive engagement between healthcare providers and patients. This comprehensive approach ensured that patients not only received adequate knowledge but also developed coping strategies and felt emotionally supported during their hospitalization.

One important consideration is the potential influence of age differences between the two groups on patient satisfaction. Previous studies have suggested that older patients often report higher satisfaction levels, possibly due to different expectations or greater appreciation for the care provided (24–26). If a significant age difference exists between the groups in our study, it could partially explain variations in satisfaction scores. However, our analysis did not find a strong correlation between age and satisfaction, suggesting that the positive impact of the intervention was not merely due to age differences but rather to the structured support provided. Future research could further explore this aspect by conducting subgroup analyses based on age to better understand its role in shaping patient satisfaction outcomes.

The findings of this study hold significant implications for clinical practice. The structured approach to patient education and psychological support could serve as a model for improving patient satisfaction in other healthcare settings. Future research should explore the potential for wider adoption of similar interventions, particularly in the management of patients with other chronic or infectious diseases. Additionally, further studies could investigate the cost-effectiveness and feasibility of implementing such programs on a larger scale.

The aforementioned studies have indicated that providing an educational package, in conjunction with regular interventions and teachings, can be effective in increasing satisfaction by addressing the needs of patients and their companions. Similarly, the results of the present study demonstrate the effective role of education in enhancing patient satisfaction.

This study encountered several limitations. The sample size was relatively small and restricted to a specific population in Najaf hospitals, which may limit the generalizability of the findings. Moreover, the study only utilized self-reported measures for patient satisfaction, which may be subject to bias. The study’s design also did not allow for long-term follow-up, preventing the assessment of the enduring impacts of the educational program. Additionally, conducting research during the COVID-19 pandemic presented unique ethical challenges, particularly regarding the process of obtaining informed consent in a high-stress hospital environment. To address these challenges, informed consent was carefully obtained while ensuring that patients fully understood the study’s purpose, procedures, and their rights, despite the stressful circumstances of hospitalization and isolation. Ethical sensitivity and adherence to patients’ autonomy and well-being were prioritized throughout the study.

Notwithstanding the limitations, the study boasts a few key strengths. The use of the well-validated Patient Satisfaction Instrument (PSI), with a high Cronbach’s alpha of 0.90, ensured the reliability of the findings. The focus on multiple dimensions of patient satisfaction—professional-technical care, trust, and education—provided a comprehensive evaluation of patient experiences. Additionally, the structured randomized clinical trial methodology enhanced the credibility of the results. However, certain limitations should be acknowledged. First, the study did not report detailed findings for the sub-scales of the PSI, which could have provided a more nuanced understanding of patient satisfaction. Second, the single-city sample limits the generalizability of the results. Lastly, the absence of long-term follow-up restricts the ability to assess the sustainability of improved satisfaction levels. Future studies should consider addressing these limitations by incorporating multi-center trials and extended follow-up periods.

Future research should consider larger, more diverse cohorts to verify and expand upon these findings. Longitudinal studies would be beneficial to examine the long-term effectiveness of supportive educational programs. It would also be pertinent to explore the individual elements of the program to determine the most effective components. Additionally, integrating objective measures of patient satisfaction, when possible, could provide a more balanced approach, and investigation into virtual delivery methods for such programs could vastly increase their reach in similar pandemic situations.

## Conclusion

The results of this investigation underline the significant effect of the supportive educational program on enhancing the satisfaction of COVID-19 patients hospitalized in Najaf. By addressing both educational and emotional support needs, the program successfully managed to alleviate symptoms of distress such as anxiety, depression, and stress, leading to higher satisfaction levels. This reinforces the notion that integrative care that includes psychological support and patient education is vital in handling pandemic outbreaks and can be a valuable addition to the standard care provided to patients with COVID-19.

## Data Availability

The raw data supporting the conclusions of this article will be made available by the authors, without undue reservation.
